# The pioneering era of bilateral internal mammary artery grafting for CABG in Europe

**DOI:** 10.3389/fcvm.2026.1776140

**Published:** 2026-02-23

**Authors:** Dritan Useini, Marcus Franz, Ralf Degenhardt, Zulfugar Taghiyev, Brigitta Gahl, Ingo Kutschka, Hassina Baraki

**Affiliations:** 1 Department of Cardiac Surgery, University Hospital Basel, University of Basel, Basel, Switzerland; 2 Department of Cardiology, Angiology and Intensive Care Medicine, Cardiovascular Center Hersfeld-Rotenburg, Rotenburg an der Fulda, Germany; 3 Institute for Clinical Research, Cardiovascular Center Hersfeld-Rotenburg, Rotenburg an der Fulda, Germany; 4 Department of Cardiac Surgery, Kerckhoff-Clinic, Campus Kerckhoff, Justus-Liebig-University of Giessen, Bad Nauheim, Germany

**Keywords:** arterial, BIMA, CABG, first European series, outcomes

## Abstract

**Objectives:**

Bilateral internal mammary artery (BIMA) grafting for coronary artery bypass surgery (CABG) was uncommon in Europe before routine coronary stenting. This study evaluates long-term outcomes from one of the earliest systematic European BIMA experiences, providing historical, epidemiological, and clinical insight.

**Methods:**

We retrospectively identified 427 patients who underwent BIMA-CABG at a single German center between 1989 and 1993. Seventy-three patients were lost to follow-up, leaving 354 for analysis. The primary endpoint was overall survival; secondary endpoints included composites of all-cause mortality, myocardial infarction (MI), stroke, and repeat revascularization. Mean follow-up was 18.4 ± 0.8 years.

**Results:**

Patients had a mean age of 57 ± 8.8 years; 88% were men. Preoperative risk factors included hyperlipidemia (78%) and smoking (71%). No patient had prior coronary stenting; 18% underwent balloon dilation or thrombolysis. Kaplan–Meier estimates a mean survival probability of 19.1 ± 0.2 years [95% confidence interval (CI) 18.6–19.6], freedom from mortality/MI/stroke 18.8 ± 0.2 years (95% CI 18.2–19.3), and from mortality/MI/revascularization 17.8 ± 0.3 years (95% CI 17.2–18.4). The overall 20-year revascularization rate was 11.6%, all treated percutaneously; no patient required repeat CABG.

**Conclusions:**

Early German BIMA-CABG, performed as first-line therapy for coronary artery disease prior to the stent era, achieved excellent 20-year survival and minimal repeat revascularization. This cohort provides unique long-term evidence from the pioneering era of arterial revascularization in Europe, highlighting clinical, epidemiological and historical significance and underscore the pioneering role of BIMA grafting in shaping modern arterial revascularization strategies.

## Introduction

The use of the bilateral internal mammary arteries (BIMA) for coronary artery bypass grafting (CABG) began in the United States and was first described by Suzuki et al. in 1973 (1). However, the widespread clinical adoption of BIMA in the United States occurred during the 1980s and early 1990s, as evidence gradually accumulated and culminated in the first major publications on the technique (2, 3). This development was based on and supported by the growing body of evidence demonstrating that the internal mammary artery provides superior long-term patency and clinical outcomes compared with the saphenous vein (4, 5).

During the same period, a new paradigm in the management of coronary artery disease (CAD) was emerging—percutaneous coronary intervention with stent implantation (6). Nevertheless, at that time, stenting had not yet become a routine therapeutic modality, and coronary artery bypass grafting (CABG) remained the standard of care for advanced CAD. Importantly, CABG practice was evolving, with the bilateral internal mammary artery (BIMA) technique offering an innovative means of achieving arterial revascularization.

In the present study, we report unique long-term outcomes of patients who underwent CABG with BIMA in Germany during the late 1980s and early 1990s—a period characterized by pioneering efforts in arterial grafting during the pre-stenting era in Europe.

## Methods

### Ethical statement

All patients provided informed consent for data collection, and approval for the study was obtained from the Ethics Committee of the Hessen State Chamber of Physicians, Frankfurt am Main; Date: 7.06. 2024; Reg. No. 2024-3658-evBO.

### Study design

This is a retrospective, single-center observational study analyzing long-term outcomes of patients who underwent CABG with BIMA grafting between 1989 and 1993. The study was conducted in accordance with the principles of the Declaration of Helsinki.

### Patient population

We identified 427 patients in our institutional archives who underwent isolated CABG with BIMA grafts for CAD between 1989 and 1993. Patients undergoing concomitant valve surgery or other cardiac procedures were excluded. Demographic data, cardiovascular risk factors, preoperative clinical status, and operative variables were collected from institutional medical records. Data from seventy-three patients were excluded due to incomplete records or loss to follow-up. Finally, 354 patients were included in the study.

### Primary and secondary endpoints

The primary end-point was long-term survival. Second end-points were: a composite of long-term freedom from all-cause mortality, myocardial infarction and repeat revascularization, a composite long-term freedom from all-cause mortality, myocardial infarction and stroke and cardiac cause of mortality.

### Surgical technique

All procedures were performed via median sternotomy under cardiopulmonary bypass with standard myocardial protection. The internal mammary arteries were harvested in a pedicle fashion. BIMA grafts were used *in-situ* for revascularization of the left anterior descending artery, right coronary artery, Ramus diagonalis, Ramus intermedius and the first obtuse marginal branch, supplemented by saphenous vein grafts as necessary for ramus intervetricularis posterior, left- and right marginal artery. The right mammary artery was tunneled through sinus transvs. if necessary. Perioperative management followed institutional protocols, including sternal wound care and antiplatelet therapy with Aspirin.

### Follow-up and outcomes

Postoperative follow-up was conducted through medical records, outpatient visits, structured telephone interviews and written interview format. The cause of death, the cardio-cerebral adverse events and the functional evaluation of deceased patients were obtained from the last physician involved in the patients’ treatment. The clinical follow-up was completed for all 354 patients. The mean follow-up time was 18.4 ± 0.8 years.

### Statistical analysis

The distributions of quantitative variables are described as means with their standard deviation (SD). The qualitative variables are summarized by count and percentage. The Kaplan–Meier method was used to calculate the median survival time. The data were managed with the SPSS statistical package (IBM SPSS Statistics for Windows, Version 23.0. Armonk, NY, USA: IBM Corp.).

## Results

### Patient characteristics

The mean age was 57 ± 8.8 years, and 88% were male**.** Common cardiovascular risk factors included hypertension (48%), diabetes mellitus (27%), hyperlipidemia (78%), and a history of smoking (71%). There were no patients with previous coronary stent implantation. Previous balloon dilation or lysis therapy underwent 18% of the patients. Three- vessel, two-vessel-, and one-vessel CAD were 75%, 23% and 2%, respectively (Table 1).

**Table 1:** Baseline characteristics.

Baseline characteristics	Study group *N* = 354 (%)
Age (years)	57 ± 8.8
Male sex	311 (88)
Hypertension	170 (48)
Diabetes	96 (27)
Hyperlipidemia	276 (78)
Smoking	251 (71)
Previous cardiac surgery	0 (0)
Previous coronary stenting	0 (0)
Previous coronary dilation or Thrombolysis	64 (18)

### Operative data

All procedures were performed via median sternotomy under cardiopulmonary bypass. Pedicle BIMA harvesting was performed in 100% of cases, with the left internal mammary artery (LIMA) typically grafted to the left anterior descending artery and the right internal mammary artery (RIMA) to right coronary artery or the circumflex territory. Supplementary saphenous vein grafts were used in 37% of patients. The mean number or BIMA anastomosis was 3 ± 1 (Table 2).

**Table 2:** Procedural and early outcomes.

Procedural and early outcomes	Study group *N* = 354 (%)
Three-vessel CAD	258 (73)
Two-vessel CAD	85 (24)
One-vessel CAD	11 (3)
On-pump CABG	354 (100)
Pedicled/in-situ BIMA	354 (100)
Supplementary saphenous vein grafts	131 (37)
30-day mortality	2 (0.6)
30-day stroke	0 (0)
30-day myocardial repeat revascularization	2 (0.6)
Wound healing disorder	11 (3)

CAD, coronary artery disease; CABG, coronary artery bypass grafting; BIMA, bilateral internal mammary arteries.

### Early outcomes

Perioperative mortality occurred in 0.6% of patients. Major early complications included sternal wound infection 3.1%, stroke 0%, and myocardial repeat revascularization 0.6% (Table 2).

### Long-Term outcomes

**Figure 1:**
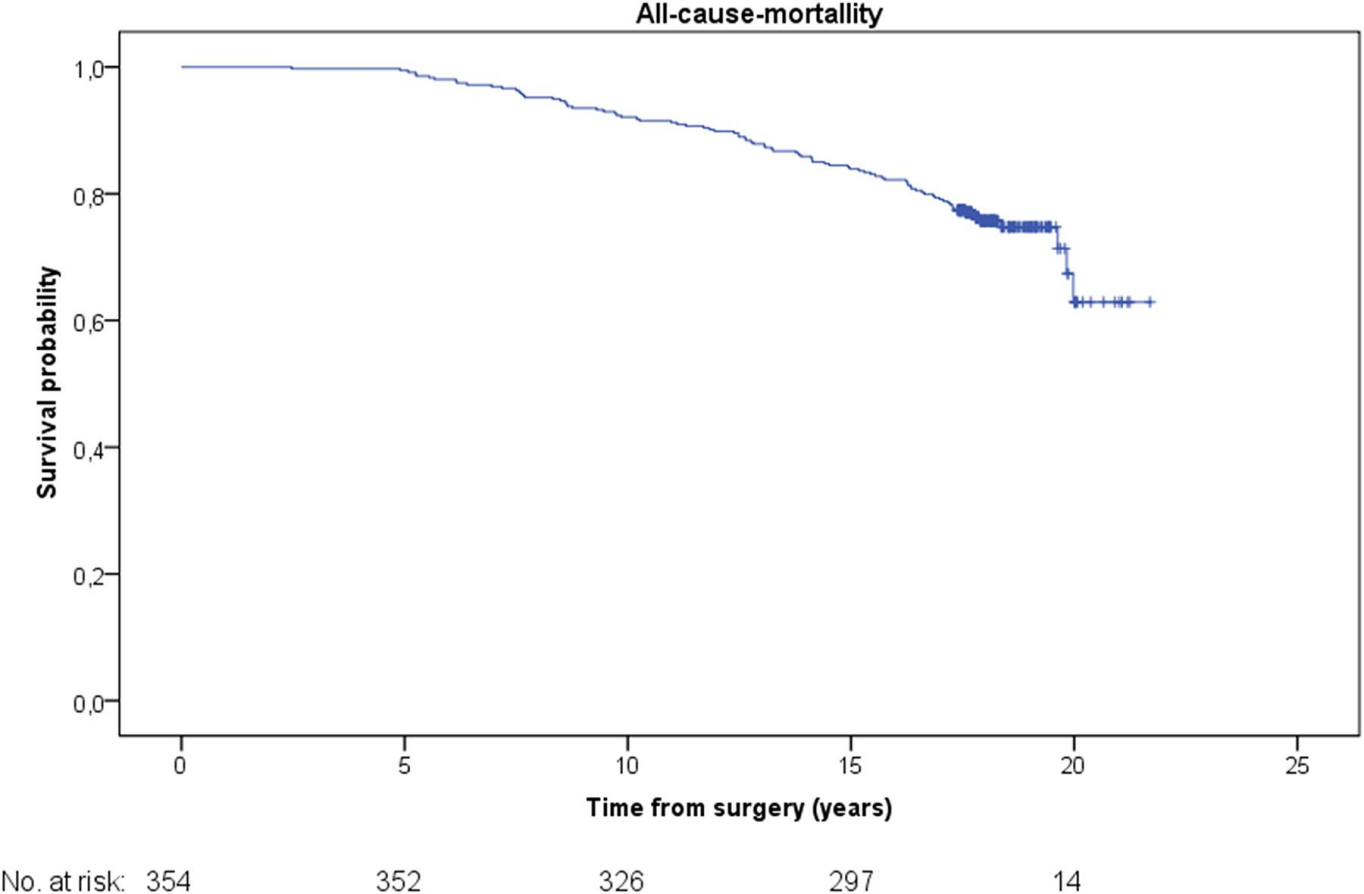
Kaplan–Meier survival function for the entire cohort.

**Figure 2:**
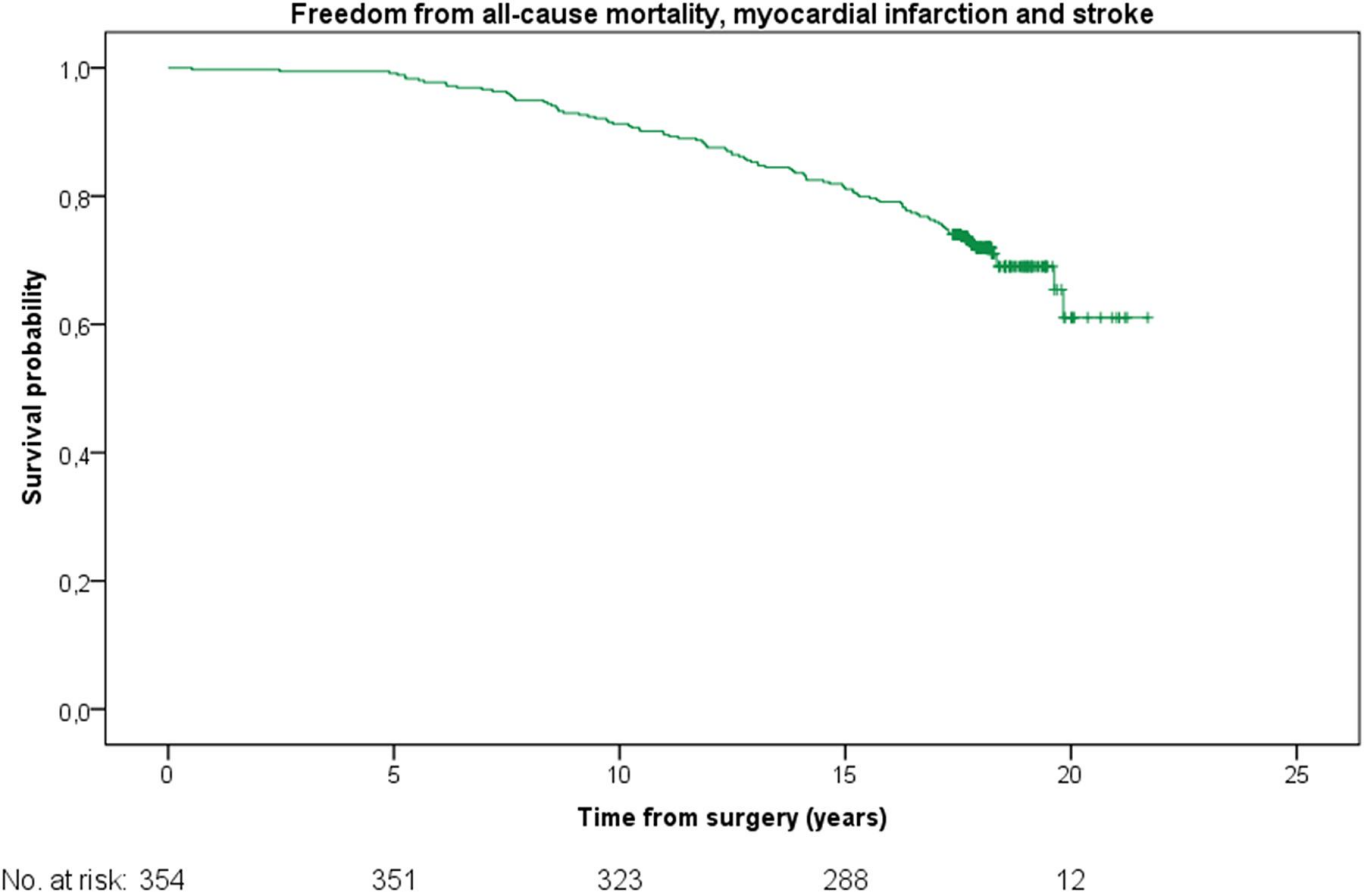
Kaplan–Meier curve: freedom from all-cause mortality, myocardial infarction and stroke for the entire cohort.

**Figure 3:**
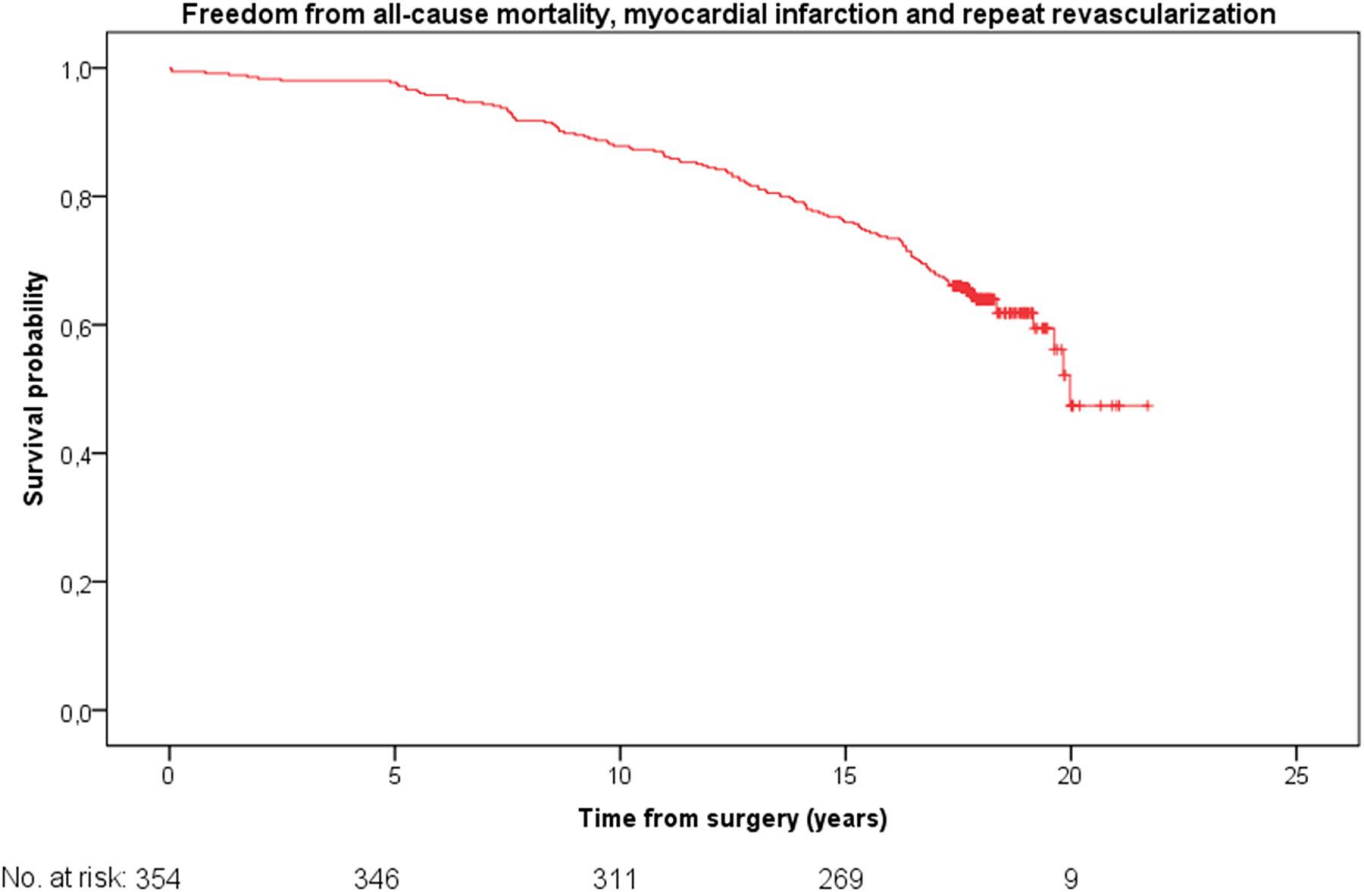
Kaplan–Meier curve: freedom from all-cause mortality, myocardial infarction and repeat revascularization for the entire cohort.

The Kaplan–Meier analysis revealed a mean survival probability of freedom from all-cause mortality was 19.1 ± 0.2 years {[95% confidence interval (CI) 18.6–19.6]} (Figure 1). The mean freedom from a composite of all-cause mortality, myocardial infarction and stroke was [18.8 ± 0.2 years (95% CI 18.2–19.3)] (Figure 2). The mean freedom from a composite of all-cause mortality, myocardial infarction and repeat revascularization was [17.8 ± 0.3 years (95% CI 17.2–18.4)] (Figure 3). The overall repeat revascularization rate after 20 years was 11.6%. No patients underwent redo surgery. All patients underwent percutaneous interventions.

### Causes of death

**Figure 4:**
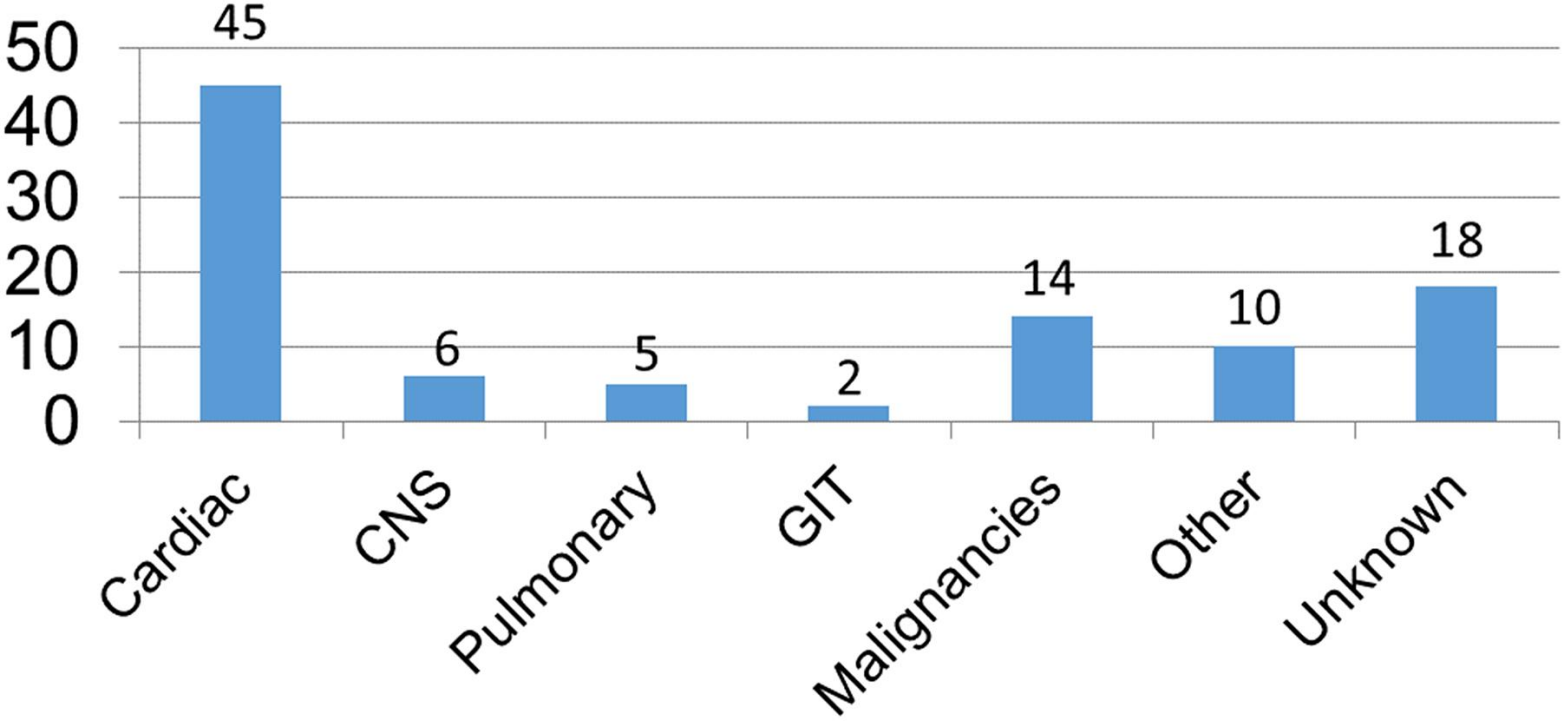
Causes of death (%).

The most common cause of death was cardiac related with a rate of 45%, followed by malignancies with a rate of 14%. Cerebral causes of death accounted for 6%. Pulmonary and gastrointestinal causes of death had rates of 5% and 2%, respectively. An unknown cause of death was registered for 18% (Figure 4).

## Discussion

The objective of this study was to evaluate the long-term clinical outcomes of patients who underwent coronary artery bypass grafting (CABG) with bilateral internal mammary artery (BIMA) grafting during the late 1980s and early 1990s at a single German institution. This cohort represents one of the earliest European experiences with systematic BIMA application prior to the advent of routine coronary stenting. By providing detailed survival and event data after more than two decades of follow-up, this analysis aims to highlight the long-term durability and clinical value of BIMA grafting and to place these findings into the historical and contemporary context of myocardial revascularization strategies.

### Main findings and historical relevance

This study documents one of the earliest systematic European experiences with bilateral internal mammary artery (BIMA) grafting for coronary artery bypass grafting (CABG) and provides over two decades of clinical follow-up—the longest clinical follow-up of initial BIMA era in Europe. The results demonstrate excellent long-term survival and freedom from major adverse cardiac events (MACE), confirming the durability of arterial revascularization with BIMA. Beyond its clinical implications, this study has substantial *historical and epidemiological relevance*: at the time these operations were performed, BIMA grafting was extremely uncommon in Europe, and few centers had adopted a structured bilateral mammary strategy.

### Context within European surgical practice

During the late 1980s and early 1990s, coronary artery bypass grafting underwent rapid development; however, the vast majority of procedures in Europe employed a single internal mammary artery (SIMA) in combination with saphenous vein grafts. Reports from that period, such as that by Angelini et al. (7), provided valuable insights into surgical practice among more than 17,000 patients in the United Kingdom but did not specifically address the use or outcomes of bilateral internal mammary artery (BIMA) grafting. The authors noted that BIMA was used only in rare cases.

Similarly, Sergeant et al. (8) reported a BIMA utilization rate of 12.4% among 9,600 patients who underwent CABG between 1971 and 1992 in Leuven, Belgium, and observed that its use was inconsistent over this more than 20-year period. Furthermore, Carrel et al. (9) published an eight-year follow-up study of CABG with BIMA performed between 1985 and 1989 in Switzerland, during which only 80 patients underwent surgery with BIMA grafting. Calafiore et al. (10) reported on BIMA use between 1991 and 2000, including an eight-year clinical follow-up. However, the incidence of BIMA use in the early 1990s remains unclear.

Consequently, the true prevalence and long-term impact of bilateral mammary revascularization in Europe remained largely undocumented.

Our data therefore fill an important historical gap. The present cohort, representing consecutive BIMA patients operated in Germany between 1989 and 1993, provides rare longitudinal evidence from a pioneering European center that systematically employed this technique well before its broader recognition.

### First-Generation BIMA and evolution to the modern multi-arterial paradigm

The BIMA strategy employed reflects a first-generation approach. Internal mammary arteries were harvested as pedicled grafts and frequently supplemented with saphenous vein grafts to achieve complete revascularization, with vein grafts used in 37% of patients. This practice reflected technical constraints of the era rather than conceptual limitations of arterial revascularization. The observed sternal wound complication rate (3.1%) must likewise be interpreted in historical context, preceding the routine adoption of skeletonized harvesting and limiting early uptake of BIMA, particularly in higher-risk patients.

Subsequent technical advances directly addressed these limitations. Skeletonized internal mammary artery harvesting reduced sternal wound complications and increased conduit flexibility (11), while the integration of the radial artery and composite grafting strategies, such as T or Y BIMA configurations (12, 13), enabled more complete arterial revascularization and reduced reliance on saphenous vein grafts (14). Combined with improved perioperative care and accumulating evidence, these refinements expanded multi-arterial grafting to older and higher-risk populations (15).

### Alignment with contemporary evidence

The long-term outcomes observed in this cohort are consistent with later observational studies and meta-analyses demonstrating a survival advantage of BIMA over SIMA grafting (16). Contemporary meta-analyses confirm that survival benefits of BIMA over SIMA extend across diverse patient subgroups (17, 18).

Recent randomized evidence from the ongoing ROMA trial provides important contemporary perspective: as the largest randomized comparison of single vs. multiple arterial grafting, ROMA is designed to evaluate long-term outcomes, but definitive results are not yet published (19, 20).

Together, these data place early BIMA experience within the continuum of coronary revascularization evolution, demonstrating the principle of arterial durability, while subsequent technical and evidence-based refinements have broadened applicability and optimized outcomes.

### Epidemiological and guideline implications

From an epidemiological perspective, this study captures a formative phase in the transition from venous to arterial coronary revascularization in Europe and highlights the role of early pioneering centers in shaping surgical practice. The durability demonstrated in this first-generation BIMA cohort aligns with current professional society recommendations, including ESC/EACTS guidelines, which support multi-arterial grafting in appropriate patients to improve long-term outcomes (21).

### Strengths and limitations

Key strengths of this study include its uniquely long follow-up and its representation of an underreported period in European cardiac surgery. Limitations include its retrospective, single-center design and the lack of contemporary comparison groups, which are inherent to historical analyses. The exclusion of 73 patients (17.1% of the original cohort) due to lost follow-up is a significant limitation for a survival study. The potential impact of this attrition on the long-term survival estimates is another limitation of the study.

## Conclusions

This study represents one of the first systematic European reports on BIMA grafting and provides valuable long-term outcome data from the pioneering era of arterial revascularization in Germany. At a time when most European CABG procedures used only one mammary artery, these results demonstrate that bilateral mammary revascularization was both feasible and durable. Beyond confirming the long-term benefits of BIMA, the study offers historical insight into the early evolution of coronary surgery in Europe and underscores the foundational contributions that anticipated today's evidence base.

## Data Availability

The raw data supporting the conclusions of this article will be made available by the authors, without undue reservation.
